# Examination of inter-rater and intra-rater reliability during retentive force measurement of different clasps using the developed small-sized retentive force measurement device

**DOI:** 10.1186/s12903-020-01215-2

**Published:** 2020-08-20

**Authors:** Hitoshi Akiyama, Maiko Sakamoto, Ryoichi Akama, Jun Takei

**Affiliations:** 1 General Dentistry, The Nippon Dental University Hospital, 2-3-16 Chiyoda-ku, Fujimi, Tokyo, 102-8158 Japan; 2 Laboratory room, The Nippon Dental University Hospital, 2-3-16 Chiyoda-ku, Fujimi, Tokyo, 102-8158 Japan

**Keywords:** Abutment tooth, Retentive force, Removable partial denture, Clasp, Intraclass correlation coefficient

## Abstract

**Background:**

The design process of a removable partial denture (RPD) consists of rests, major connectors, minor connectors, denture base, and retainer. The abutment tooth contour determines the location of the retention portion of the clasp at the retention areas. The load capacity of the clasp depends on various factors such as type, the position of a clasp, tooth position, clasp length, and pullout location. As a general rule, the amount of retention required to dislodge the RPD from the supporting structure should always be the minimum necessary to resist reasonable dislodging forces. Excessive force from the clasps may cause many problems. Although there are many reports on the retentive force of cast clasps using large devices, it has not been possible to measure it using simple devices until now.

**Method:**

The purpose of the present study is to develop a small-sized retentive force measurement device that can easily measure the retentive force of a clasp used for an RPD. It is to examine inter-rater and intra-rater reliability. A small-sized retentive force measurement device that can be easily measured in the laboratory has been developed. Using commercially available hard plaster cast, a skilled dental technician has made 10 types of cast clasps used in clinical practice using conventional techniques. Three assessors measured the retentive force of 10 types of cast clasps. To confirm the reliability, the intra-class correlation coefficients ICC (1,1) and ICC (1,3) of the 3 assessors were calculated, and the reliability within the assessor was examined. The inter-class correlation coefficients ICC (3,1) and ICC (3,3) were calculated, and the reliability of the assessors was examined.

**Result:**

The intra-class correlation coefficients of 3 assessors are as follows: assessor 1 has ICC (1,1) = 0.971, ICC (1,3) = 0.990, assessor 2 has ICC (1,1) = 0.967, ICC (1,3) = 0.989, assessor 3 has ICC (1,1) = 0.962, ICC (1,3) = 0.987. The inter-class correlation coefficients of 3 assessors are as follows: ICC (3,1) = 0.993, ICC (3,3) = 0.998. From the evaluation standard of the intraclass correlation coefficients of reliability value by ICC, it was evaluated as almost perfect and high reproducibility was confirmed.

**Conclusion:**

The developed small-sized retentive force measurement device has reproducibility within and between the assessors.

## Background

In a treatment aiming for the functional recovery of a partially edentulous patient’s masticatory system, intraoral installment of prosthetic devices functioning as artificial organs are often used. Removable partial denture (RPD) are widely used to replace missing teeth and lost alveolar tissue, thereby restoring aesthetic and function [1]. RPD should be esthetic with minimum periodontal tissue problem and sufficiently retentive to avoid begin displaced function. From the design of RPD considering occlusal pressure distribution, the design process of RPD consists of rests, major connectors, minor connectors, denture base, and retainers. The load capacity of the clasp depends on various factors such as type, the position of a clasp, tooth position, clasp length, and pullout location. The abutment tooth contour determines the location of the retention portion of the clasp at the retention areas [2]. The clasp of RPD is generally made of 0.25 mm, 0.50 mm, and 0.75 mm undercuts for near and far zones [3]. As a general rule, the amount of retention required to dislodge the RPD from the supporting structure should always be the minimum necessary to resist reasonable dislodging forces. Excessive force from the clasps may cause many problems [4]. Circumferential clasps are the most frequently used direct retainers, and long term success of RPD depends on properties of clasps [5]. The retentive force is the force that works when the RPD is removed, and an appropriate force must be applied to the abutment tooth. Unfortunately, an inadequate denture design or retainer often generates an excessive retentive force to cause the movement of abutment tooth, thereby resulting in the mobility of abutment tooth. Additionally, this increases the abutment tooth’s load, and eventually, the abutment tooth is lost. Inadequate use of RPD must be avoided so as not to miss a natural tooth, an integral part of a human body.

Regarding the measurement of the retentive force of the clasp for RPD, there are also several studies on clasps using the finite element method [6], on measuring the retentive force using a universal testing machine with a crosshead speed [7], on weighing the retentive force of removal and insertion cycling of clasp [8], on cyclic fatigue properties of alloy cast clasps [9], on an evaluation of retentive ability and deformation [10], on the comparison of buccal and lingual retention [11], and on clasps compared with the metal materials used [12]. These are all studies using large-scale experimental equipment, and until now, there was no equipment in the dental field that could easily measure the retentive force of a clasp of RPD.

In the present study, it is significant to present a retentive force measurement device that can be easily used in the laboratory to provide an RPD with adequate retentive force.

The purpose of the present study is to develop a small-sized retentive force measurement device that can easily measure the retentive force of a clasp used for an RPD, and it is to examine the inter-rater and intra-rater reliability.

## Methods

### Development of the small-sized retentive force measurement device

The small-sized retentive force measurement device has a structure that detects force as a bending moment. Processing was carried out to the side where there was no chip at the tip of a commercially available band removing plier (band-removing pliers for molars 60–104, Task Co., Ltd., Tokyo, Japan). Two strain gauges (gauge length of 1 mm and base size of 1.4 × 2.8 mm, Kyowa Electronic Instruments Co., Ltd., Tokyo, Japan) for transducers were provided inside and two were given to the outside, and a cable with 1.7 calibers was welded from the terminal of the gauge towards the handles. Strain gauge has characteristics such as excellent repeatability and linearity, and it is possible to measure force, load, pressure, and displacement [13, 14]. After the coating process, the device was covered with silicon rubber. For improvement of reproducibility, four-active-gauge method (torsion strain measurement method) was adopted, strain gauge bridge was assembled at four places, and the difference of strain generated at the four locations was output. We reduced the output error due to the difference in the loading point.

By outputting the difference between strain detection positions L1 and L2, ε = F · (L1 − L2) / (Z · E).

(ε: strain output, Ơ: stress, F: force, Z: section modulus, E: Young’s modulus, M: bending moment, L: distance)

**Fig. 1:**
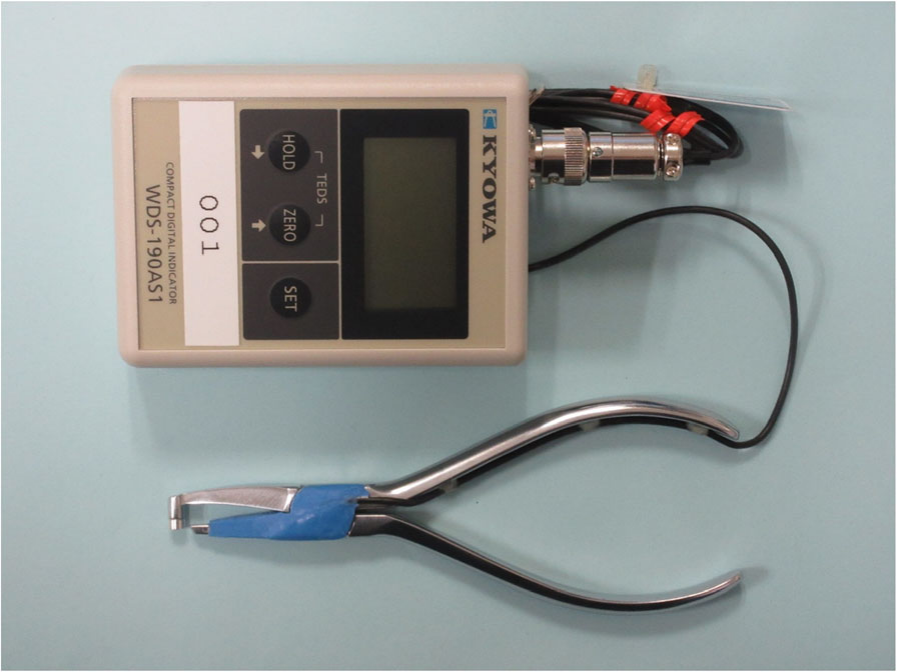
The developed small-sized retentive force measurement device

**Fig. 2:**
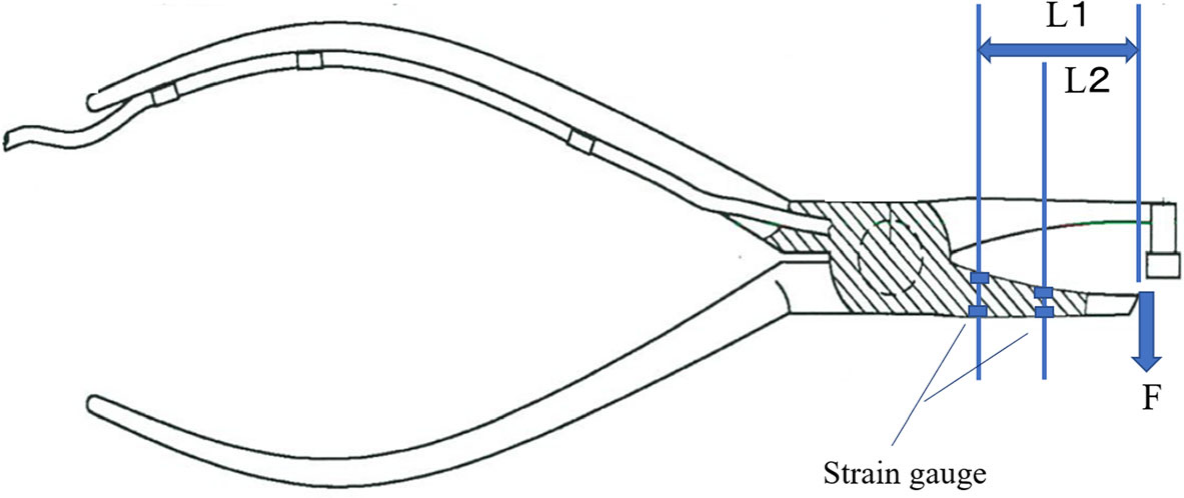
The structure schematic of the developed small-sized retentive force measurement device

Subtracting L2 from L1 is always invariable irrespective of the position of the load point; the structure was such that the magnitude of the load F could be accurately measured. For the sensor conditioner, the present study used a compact digital indicator (Kyowa Electronic Instruments Co., Ltd. WDS-190AS1, Tokyo, Japan) to quantify the retentive force of various clasps. The amount of distortion at the time of measurement can be converted and displayed as a retentive force. The output sensitivity was fixed at about 500 × 10^− 6^ strain/1 kgf. The maximum peak value of the retentive force is presented on display by applying the shorter side of the small-sized retentive force measurement device attached with strain gauges on the lower arm of the retention arm of the undercut area of the retainer affixed on the abutment tooth and by vertically applying the flat-tipped edge on the occlusal surface of the abutment tooth. Figure 1 shows the developed small-sized retentive force measurement device. Figure 2 shows the structural schematic of the developed small-sized retentive force measurement device.

### Change to device calibration

The strain level is measured using a measuring instrument (Kyowa Electronic Instruments Co., Ltd. PCD-300B, Tokyo, Japan) to measure the strain value of the plier gauge when each load is applied to the strain sensing area of the plier of the small-sized retentive force measurement device. To confirm whether the measurement using the small-sized retentive force measurement device can be done appropriately, the strain levels (με) were measured under loads of 0 g, 100 g, 200 g, 400 g, 800 g, 1200 g, 1600 g, and 2000 g.

### Production of 10 types of cast clasps

The 10 types of cast clasps were as follows according to the removable partial prosthodontics of McCracken [15].

**Fig. 3:**
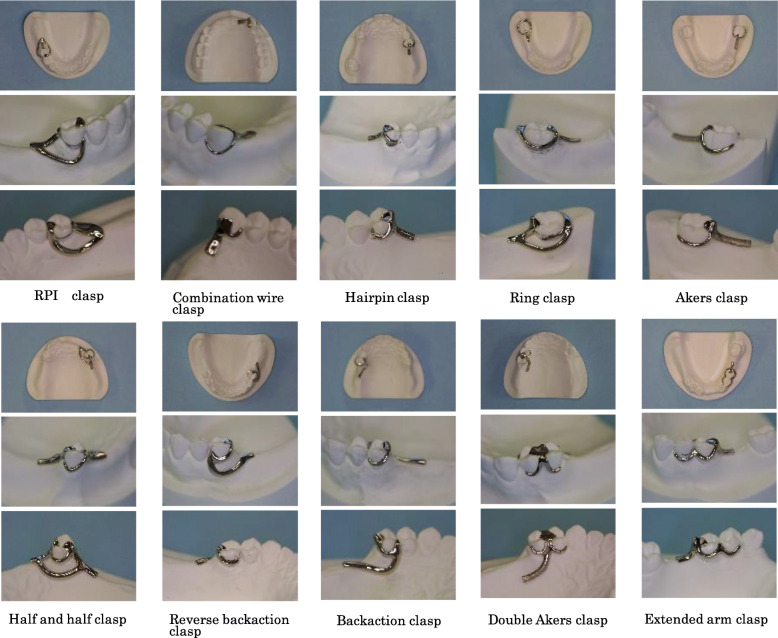
10 types of cast clasps used in the present study

An RPI clasp and a combination clasp using 0.25 mm undercut, a hairpin clasp, a ring clasp, an Akers clasp, a half and half clasp, a reverse backaction clasp, a backaction clasp, a double Akers clasp, and an extended arm clasp using the 0.50 mm undercut (Fig. 3).

Using commercially available hard plaster cast, a skilled dental technician has made 10 types of cast clasps used in clinical practice using conventional techniques. Regarding the production of 10 types of cast clasps, a pre-made wax pattern for cast clasp (wax pattern MK 110–002-00, Dentaurum Co., Ltd., Osaka, Japan) was used for cast clasp with undercut depth at 0.5 mm. A dental casting gold and silver palladium alloy (Kimpara G12, Ishihuku Metal Industry Co., Ltd., Tokyo, Japan) commonly used in dental practice in Japan were employed. Confirmation of conformity state of cast clasp was carried out using a high spot indicator (Arti-Spot®, Bausch Occlusion Paper Japan, Osaka, Japan).

### Retentive force measurement with the small-sized retentive force measurement device

Three assessors measured the retentive force of 10 types of cast clasps and had more than 5 years of clinical experience.

**Fig. 4:**
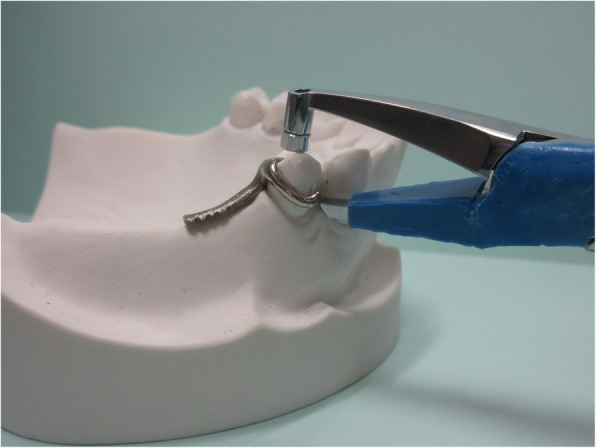
The measurement situation of the small-sized retentive force measurement device

When the handle of the measurement device is closed so that the crosshead speed becomes constant, the retention arm slightly lift-up, and the separation force generated at that time is measured. Figure 4 shows the measurement situation of the small-sized retentive force measurement device.

### Statistical analysis

Statistical analysis was conducted with analytical software PASW Statistics 18 (SPSS, IBM Co., Tokyo, Japan).

Intraclass correlations coefficient (ICC), one-way, two-way random effects model, ICC with 95% confidence interval (Cl) were used to measure the inter-intra rater reliability for the quantitative measurements.

To confirm the reliability within the assessor, the intra-class correlation coefficients ICC (1,1) and ICC (1,3) of the 3 assessors were calculated. Further, to confirm the reliability between the assessors, the inter-class correlation coefficients ICC (3,1) and ICC (3, 3) were calculated from the average of the measured values of the 3 assessors [16].

## Results

### Relationship between load and strain amount by the small-sized retentive force measurement device

**Fig. 5:**
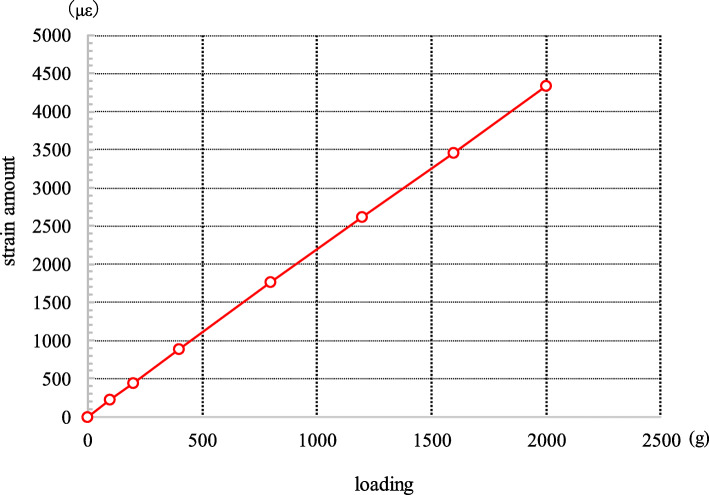
Relationship between load and strain amount by the small-sized retentive force measurement device

The strain levels (με) measured by the small-sized retentive force measurement device when loads of 0 g, 100 g, 200 g, 400 g, 800 g, 1200 g, 1600 g, and 2000 g were respectively added showed a straight-line increase, which can be translated into a linear function (calibration constant: 0.4610 g/1 με) (Fig. 5).

### Retentive force measurement using the small-sized retentive force measurement device

Table 1 shows the results of the retentive force measurement of 10 types of cast clasps of 3 assessors. The intra-class correlation coefficients of 3 assessors are as follows: assessor 1 has ICC (1,1) = 0.971, ICC (1,3) = 0.990, assessor 2 has ICC (1,1) = 0.967, ICC (1,3) = 0.989, and assessor 3 has ICC (1,1) = 0.962, ICC (1,3) = 0.987 (Table 2). From the evaluation standard of the intra-class correlation coefficients of reliability value by ICC [17], it was evaluated as almost perfect, and high reproducibility was confirmed. To confirm the reliability between 3 assessors, the inter-class correlation coefficients calculated from the average value of the measured values of the 3 assessors was ICC (3,1) = 0.993, ICC (3,3) = 0.998 (Table 3). From the evaluation standard of the inter-class correlation coefficients of reliability values by ICC [17], it was evaluated as almost perfect, and it was found that the reliability between assessors was high.

**Table 1:** The results of retentive force measurement of 10 types of cast clasps

10 types of cast clasps	Model number (Nissin Co.Ltd)	Undercut amount	Assessor 1			Assessor 2			Assessor 3		
			1st time(g)	2nd time(g)	3rd time(g)	1st time(g)	2nd time(g)	3rd time(g)	1st time(g)	2nd time(g)	3rd time(g)
RPI clasp	E3-522	0.25mm	262.4	218.5	239.3	197.3	282.6	230.8	273.7	230.6	245.2
Combination wire clasp	E3-583	0.5mm	291.4	235.6	310.2	256.9	270.6	217.8	334.3	295.2	265.3
Hairpin clasp	E3-545	0.5mm	530.7	513.4	464.5	517.4	542.1	616.4	584.2	489.3	510.7
Ring clasp	E3-534	0.5mm	632.4	645.3	680.4	664.6	632.5	685.5	673.2	659.2	599.2
Akers clasp	E3-541	0.5mm	737.4	659.5	630.5	690.4	630.2	604.7	659.3	690.6	722.4
Half and half clasp	E3-563	0.5mm	752.3	680.5	690.8	732.3	680.4	713.2	650.2	732.3	677.3
Reverse backaction clasp	E3-522	0.5mm	720.5	740.5	710.3	650.3	708.5	743.2	688.4	751.3	707.3
Backaction clasp	E3-567	0.5mm	670.3	732.7	765.4	703.5	763.2	740.2	747.3	688.6	770.2
Double Akers clasp	E3-530	0.5mm	898.2	810.4	880.6	840.2	862.5	780.3	821.3	879.3	760.1
Extended arm clasp	E3-546	0.5mm	929.3	870.4	860.4	880.2	924.4	826.3	931.3	889.3	834.2

**Table 2:** Intra-rater reliability of the results of retentive force measurement of 3 assessors

	ICC (1,1)	95%Cl	ICC (1,3)	95%Cl
assesor 1	0.971	0.920-0.992	0.990	0.972-0.997
assesor 2	0.967	0.911-0.991	0.989	0.968-0.997
assesor 3	0.962	0.896-0.989	0.987	0.963-0.996

*ICC* Intraclass correlation coefficient, *95%Cl* 95% Confidence interval (lower-upper)

**Table 3:** Inter-rater reliability of the results of retentive force measurement among assessors

	ICC (3,1)	95%Cl	ICC (3,3)	95%Cl
3 assesors	0.993	0.980-0.998	0.998	0.993-0.999

*ICC* Intraclass correlation coefficient, *95%Cl* 95% Confidence interval (lower-upper)

## Discussion

In principle, the effect of support and bracing functions when wearing a partial denture. The impact of retention does not act during wearing a partial denture because the retentive force acts on the retentive arm that has entered the undercut when detaching the partial denture. When wearing a partial denture, the clasp closely conforms to the tooth surface and should not exert any force on the tooth. No burden on the abutment tooth is observed and prevents detachment of the denture. The retentive force of a clasp works only when the denture is removed. To date, the retentive force of a clasp has been reported in many laboratory studies [6–12, 18–26]. Ahmad et al. [18] mentioned that the mean retentive force for a framework engaging an undercut of 0.25 mm with Akers clasp was 4.77 N. Meenakshi et al. [10] showed the clasp required 7.24 N for 0.25 mm undercut and 8.37 N for 0.50 mm undercut. Frank and Nicholls [19] concluded that 300 to 750 g (2.94 to 7.35 N) represented an acceptable amount of retention for a bilateral distal extension RPD. Arda and Arikan [20] tested the retentive force of cast Akers clasp was 615.2 ± 14.5 g with the undercut depth at 0.25 mm, 858.2 ± 58.2 g with the undercut depth at 0.5 mm. Bridgeman et al. [21] showed retentive forces from 5 N to 10 N would be necessary in one clasp. Wie et al. [22] showed the initial retentive force of the clasps was higher than 5 N, the final retentive force of the 0.50 mm undercut clasps was approximately 5 N. de Torres et al. [23] showed mean retentive force of RPD clasp was 8.09 ± 3.05 N with Co-Cr circumferential clasps. Tse et al. [24] found that retentive forces of Co-Cr clasps at undercut depths of 0.25 mm, 0.50 mm, and 0.75 mm were 2.34 ± 0.23 N, 4.65 ± 0.35 N, and 7.56 ± 0.50 N, respectively. It has been reported that the retentive force varies depending on the difference in modulus of elasticity of metal used for clasp [25]. The mechanical properties of 12% Au-Pd alloy and Co-Cr alloy are Vickers hardness of 280 and 365, and tensile strength of 804 Mpa and 938 Mpa, respectively [26]. Although there are differences in the metals used, these numerical data are similar to the measurements of the retentive force of the clasp of the present study using the small-sized retentive force measurement device.

Intraclass correlation coefficients are used in reliability studies [16, 17, 27–29]. In the present study, intraclass correlation coefficients were used to examine whether there was inter-rater reliability and intra-rater reliability when measuring the retentive force of 10 types of cast clasps. The standard of intraclass correlation coefficients is 0.0 to 0.20 for slight, 0.21 to 0.40 for fair 0.41 to 0.60 for moderate, 0.61 to 0.80 for substantial, 0.81 to 1.00 for almost perfect [17]. ICC (1,1) indicates the intra-rater reliability when one evaluator makes multiple evaluations. ICC (1,3) indicates the reliability of the average value when one evaluator makes multiple evaluations. As a result of the present study, ICC (1,1) and ICC (1,3) of 3 assessors showed 0.9 or more. Accordingly, it was confirmed that the reproducibility within the assessor of this measurement device was high. ICC (3,1) indicates the inter-rater reliability when multiple evaluators evaluate once. ICC (3,3) indicates the reliability of the average evaluation value when multiple evaluators evaluate once. As a result of the present study, ICC (3,1) and ICC (3,3) of 3 assessors were both 0.9 or more, and it was found that the reliability between assessors was high. Thus, it was confirmed that the retentive force measurement by the developed small-sized retentive force measurement device has high reliability within and between the assessors.

By using the developed small-sized retentive force measurement device, it is possible to observe the retentive force of the retainers manufactured on the technician side. It will be possible to easily measure the retentive force of RPD in vitro.

### Limitations

The present study is an in vitro study, and the developed small-sized retentive force measurement device of the present study measures the peeling force generated when the retentive arm is slightly lifted. This device measures the retentive force of one clasp. The RPD has several clasps, and when removing the RPD, several clasps can be lifted simultaneously in the removal direction. The device lifts the retentive arm slightly so that it can be used successfully with dentures with multiple clasps. Measurement with actual dentures is a future task.

## Conclusions

The present study successfully developed a small-sized retentive force measurement device that enables a secure measurement of the retentive force of various clasps applied to RPDs. From examining the intraclass correlation coefficients, it was confirmed that the developed small-sized retentive force measurement device has reproducibility within and between the assessors.

## Data Availability

The datasets used and/or analyzed during the current study are available from the corresponding author on reasonable request.

## Abbreviations

ICC: Intraclass correlation coefficient
Cl: Confidence interval
RPD: Removable partial denture
